# Quinacrine Ameliorates Cisplatin-Induced Renal Toxicity via Modulation of Sirtuin-1 Pathway

**DOI:** 10.3390/ijms221910660

**Published:** 2021-10-01

**Authors:** Nada F. Abo El-Magd, Hasnaa Ali Ebrahim, Mohamed El-Sherbiny, Nada H. Eisa

**Affiliations:** 1 Biochemistry Department, Faculty of Pharmacy, Mansoura University, Mansoura 35516, Egypt; 2 Department of Basic Medical Sciences, College of Medicine, Princess Nourah Bint Abdulrahman University, Riyadh 11564, Saudi Arabia; haebrahim@pnu.edu.sa; 3 Department of Anatomy, Faculty of Medicine, Mansoura University, Mansoura 35516, Egypt; 4 Department of Basic Medical Sciences, College of Medicine, AlMaarefa University, Riyadh 13713, Saudi Arabia; msharbini@mcst.edu.sa

**Keywords:** quinacrine, SIRT-1, nephrotoxicity, inflammation, apoptosis

## Abstract

Renal toxicity is a serious side effect that hinders the use of cisplatin, a commonly used and effective chemotherapeutic agent. Meanwhile, quinacrine is an FDA approved drug that has been stated for its anti-inflammatory effect. Thus, we investigated the ameliorative effect of quinacrine against cisplatin-induced renal toxicity. Single intraperitoneal (i.p.) 10 mg/kg cisplatin administration induced renal injury in rats. Our results showed that 10 mg/kg/day quinacrine decreased the mortality rate of rats from 46.15% (cisplatin group) to 12.5%, and significantly decreased renal tissue fibrosis, relative kidney to body weight ratio, serum creatinine and urea levels compared with the cisplatin group. Indeed, quinacrine significantly decreased renal malondialdehyde concentration and increased renal total antioxidant capacity, compared with the cisplatin group. Furthermore, quinacrine caused significant upregulation of renal sirtuin-1 (SIRT-1) with significant downregulation of intercellular adhesion molecule-1 (ICAM-1) and tumor necrosis factor-α (TNF-α). Moreover, quinacrine significantly blocked cisplatin-induced apoptosis, which was made evident by downregulating renal apoptotic proteins (BAX and p53) and upregulating the renal anti-apoptotic protein BCL2, compared with the cisplatin group. In conclusion, this study demonstrates, for the first time, that quinacrine alleviates cisplatin-induced renal toxicity via upregulating SIRT-1, downregulating inflammatory markers (ICAM-1 and TNF-α), reducing oxidative stress, and inhibiting apoptosis.

## 1. Introduction

Cisplatin is an efficient chemotherapeutic agent in in the treatment of different types of cancers, including non-small cell lung carcinoma, cervical, ovarian, testicular and head and neck cancers [1,2,3]. Unfortunately, cisplatin clinical use is accompanied with serious side effects on various normal tissues [4]. Cisplatin accumulates in renal proximal tubules, causing acute kidney injury [5]. Approximately 50% of cancer patients undergoing cisplatin chemotherapy suffer from renal dysfunction [6]. Hence, finding an agent that alleviates cisplatin-induced renal injury is of great importance for cisplatin-treated cancer patients.

It has been shown that cisplatin induces renal injury via increasing oxidative stress levels, mitochondrial dysfunction, inflammation, DNA damage and apoptosis [7]. Sirtuin-1 (SIRT-1), a NAD^+^ dependent histone deacetylase, has recently gained considerable attention, due to its differential regulation of inflammation, stress response and apoptosis [8,9]. SIRT1 has been shown to play a key role in cisplatin-induced apoptosis via deacetylation of p53 [10,11]. Thus, SIRT-1 represents an attractive therapeutic target for inflammatory-mediated renal injuries [9,12].

Quinacrine is an FDA-approved antimalarial drug and a well-known anti-inflammatory agent. It has also been reported to be beneficial in managing cancer, lupus erythematosus, and cutaneous sarcoidosis [13,14,15]. Mechanistically, several studies have reported the link between quinacrine and inflammation via p53 and superoxide dismutase activation as well as NF-κB and phospholipase A2 inhibition. However, no studies have investigated the role of quinacrine in alleviating cisplatin-indued renal damage via regulation of the SIRT-1 pathway. Consequently, in the current study, we hypothesize that quinacrine might alleviate cisplatin-induced acute renal injury via differential regulation of the SIRT-1/inflammatory/apoptotic axis.

## 2. Results

### 2.1. Quinacrine Attenuated Cisplatin-Induced Mortality, Nephrotoxicity and Oxidative Stress

**Figure 1:**
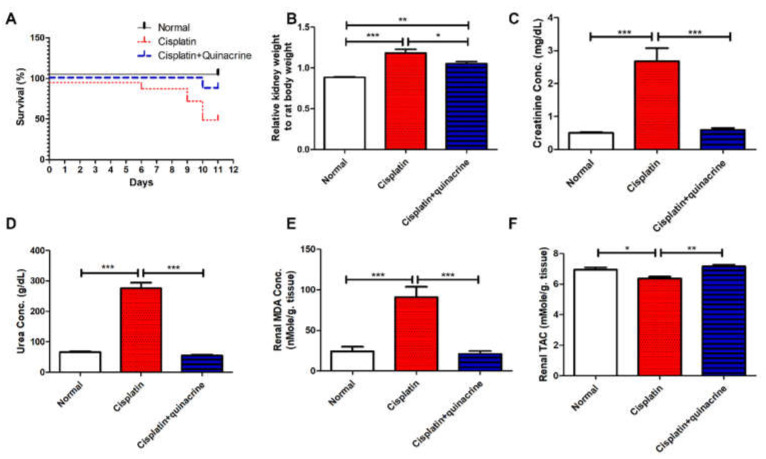
Quinacrine attenuated cisplatin-induced mortality, nephrotoxicity and oxidative stress. Effect of 10 days’ administration of quinacrine (10 mg/kg/rat/ day, intraperitoneal) against cisplatin-induced nephrotoxicity in rats on (**A**) % survival (*n* = 7–8), (**B**) relative kidney to rat body weight ratio (*n* = 7–8), (**C**) serum creatinine concentration, (**D**) serum urea concentration (*n* = 7–8), (**E**) renal malondialdehyde (MDA) concentration (*n* = 7–8) and (**F**) renal total antioxidant capacity (TAC) (*n* = 7–8). *: *p* < 0.05; **: *p* < 0.01 and *** *p* < 0.001. Data are expressed as mean ± SEM.

Cisplatin induced mortality in 46.2% of rats (Figure 1A) along with inducing a significant increase in the relative kidney to body weight ratio (Figure 1B), serum creatinine (Figure 1C), and urea (Figure 1D) concentrations, compared to the normal group. Quinacrine protected rats from these nephrotoxic effects as indicated by decreasing the mortality rate to 12.5% (Figure 1A). Moreover, quinacrine caused a significant decrease in the relative kidney to rat body weight ratio (Figure 1B), serum creatinine (Figure 1C) and urea (Figure 1D) concentrations, compared with the cisplatin group.

Cisplatin induced oxidative stress in renal tissues, as it significantly increased renal malondialdehyde (MDA) concentration (Figure 1E) and significantly decreased total antioxidant capacity (TAC) (Figure 1F), compared to the normal group. Quinacrine protected against cisplatin-induced oxidative stress, as it significantly decreased renal MDA concentration (Figure 1E) and significantly increased TAC (Figure 1F), compared with the cisplatin group.

### 2.2. Quinacrine Attenuated Cisplatin-Induced Renal Structure Alteration and Fibrosis

**Figure 2:**
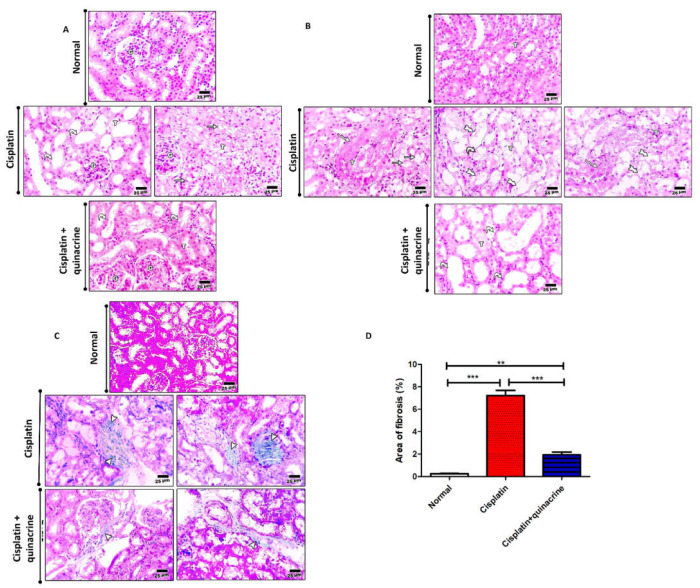
Quinacrine attenuated cisplatin-induced renal structure alteration and fibrosis. (**A**) Microscopic pictures of H&E-stained renal sections showing normal cortex including glomeruli (G) and tubules (T) in normal group, tubular dilation lined with flattened epithelium (curved arrows), vacuolar degenerative (arrows) in cortex of cisplatin group and mild tubular dilation lined with cuboidal epithelium (curved arrows) in cortex of cisplatin + quinacrine treated group. (**B**) Microscopic pictures of H&E-stained renal sections showing normal medulla cortex in normal group, marked tubular dilation (curved arrows), hydropic degeneration (rocket arrows) and necrosis (wavy arrows) in medulla in cisplatin group and moderate tubular dilation (curved arrows) in medulla of cisplatin + quinacrine group. (**C**) Microscopic pictures of Masson trichrome stained renal sections showing no fibrosis in normal group, bluish green fibrous tissue deposition (arrowheads) in renal sections from cisplatin group and the fibrous tissue deposition markedly decreased in cisplatin + quinacrine group. High magnification X: 400 bar 25. (**D**) The effect on area of fibrosis (%) of quinacrine administration (10 mg/kg/rat/ day: IP) against cisplatin-induced nephrotoxicity in rats (*n* = 6). **: *p* < 0.01 and *** *p* < 0.001. Data are expressed as mean ± SEM.

Cisplatin induced tubular dilation lined with flattened epithelium and vacuolar degeneration in the renal cortex (Figure 2A), and marked tubular dilation, hydropic degeneration, and necrosis in renal medulla (Figure 2B). Moreover, cisplatin significantly increased the area of fibrosis (Figure 2C,D), compared to the normal group. Quinacrine attenuated cisplatin-induced fibrosis and structural changes. This was evident by mild tubular dilation lined with flattened epithelium in the renal cortex (Figure 2A), moderate tubular dilation in the renal medulla (Figure 2B), and significant decrease in the percentage of fibrotic area (Figure 2C,D), compared to the cisplatin group. Although the quinacrine treatment greatly improved renal structural organization compared to the cisplatin group, it could not completely reverse cisplatin-induced renal degeneration (Figure 2A,B).

### 2.3. Quinacrine Attenuated Cisplatin-Induced Dysregulation of SIRT-1, ICAM-1 and TNF-α

**Figure 3:**
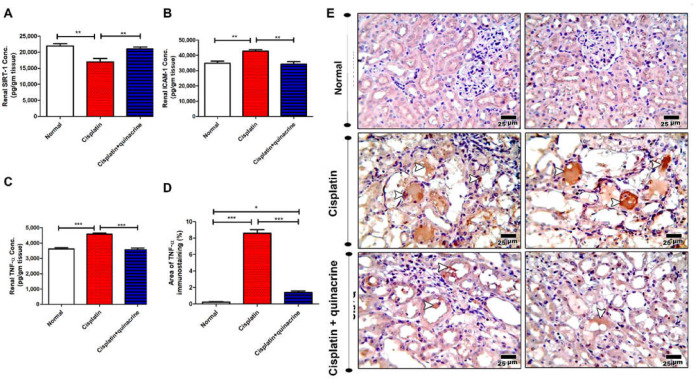
Quinacrine attenuated cisplatin-induced dysregulation of SIRT-1, ICAM-1 and TNF-α. Effect of quinacrine administration (10 mg/kg/rat/ day: i.p) against cisplatin-induced nephrotoxicity in rats on (**A**) renal sirtuin-1 (SIRT-1) concentration (*n* = 7–8), (**B**) renal intercellular adhesion molecule-1 (ICAM-1) concentration (*n* = 7–8), (**C**) renal tumor necrosis factor-α (TNF-α) concentration (*n* = 7–8) and (**D**) % area of TNF-α immunostaining (*n* = 6). (**E**) Microscopic pictures of immunostained renal sections against TNF-α showing negative expression in normal group and strong positive tubular expression appears in affected areas in renal sections from cisplatin group. The positive brown tubular expression decreased in renal sections from cisplatin + quinacrine group. (Arrowheads point to positive brown reaction.) High magnification X:400 bar 25. *: *p* < 0.05; **: *p* < 0.01 and *** *p* < 0.001. Data are expressed as mean ± SEM.

Cisplatin significantly decreased renal SIRT-1 concentration (Figure 3A) and significantly increased renal intercellular adhesion molecule-1 (ICAM-1) concertation (Figure 3B), compared with the normal group. Moreover, cisplatin significantly increased renal tumor necrosis factor-α (TNF-α) concentration (Figure 3C–E), compared to the normal group. Interestingly, quinacrine successfully attenuated cisplatin-induced dysregulation of the above-mentioned markers. Quinacrine significantly upregulated renal SIRT-1 concentration (Figure 3A), and significantly downregulated renal ICAM-1 (Figure 3B) and TNF-α (Figure 3C–E) concentrations, compared to the cisplatin group.

### 2.4. Quinacrine Attenuated Cisplatin-Induced Apoptosis

**Figure 4:**
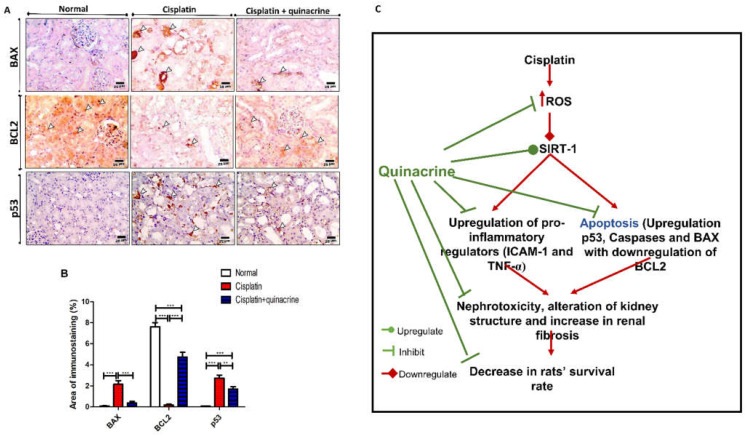
Quinacrine attenuated cisplatin-induced increase in apoptotic proteins and decrease in antiapoptotic protein. (**A**) Microscopic pictures of immunostained renal sections against BAX, BCL2 and p53; arrowheads point to positive brown reaction; high magnification X:400 bar 25. (**B**) Area of immunostaining of BAX, BCL2 and p53 (%) (*n* = 6). **: *p* < 0.01, ***:*p* < 0.001. (**C**) Schematic diagram showing potential pathway of quinacrine’s renoprotective effect against cisplatin-induced toxicity. Data are expressed as mean ± SEM.

Cisplatin caused apoptosis in renal tissue, which was indicated by the significant increase in immunostaining of apoptotic proteins BAX and p53 (Figure 4A,B), compared to the normal group. Additionally, immunostaining demonstrates that cisplatin significantly decreased the concentration of the anti-apoptotic protein BCL2 (Figure 4A,B), compared to the normal group. On the other hand, quinacrine significantly decreased the immunostaining area of apoptotic proteins BAX, p53 and significantly increased immunostaining area of anti-apoptotic protein BCL2 (Figure 4A,B), compared to the cisplatin group. These results are in accordance with the effect of cisplatin and quinacrine on caspases 1,3,8 and 9 (Supplementary Figure S1).

## 3. Discussion

Nephrotoxicity is a serious side effect of cisplatin that limits its clinical use. This study aims to investigate the potential protective effect of quinacrine, an FDA-approved drug, against cisplatin-induced nephrotoxicity, in addition to studying the possible underlying molecular mechanism of quinacrine’s action.

Single injection of cisplatin in rats induced nephrotoxicity, which was evident by the increase in mortality rate, relative kidney to body weight ratio, serum creatinine and urea concentrations, fibrosis formation and structural alterations of renal cortex and medulla. This was in agreement with El-Sherbiny et al. [16]. Moreover, cisplatin treatment led to a significant increase in renal MDA concentration and a decrease in TAC, which indicates cisplatin-induced oxidative stress in renal tissue. These results were consistent with previous studies that reported similar outcomes of cisplatin toxicity [17,18,19]. Quinacrine efficiently reversed cisplatin-induced nephrotoxicity phenotype. Previous studies show similar protective effects of quinacrine against in vivo cyclosporine-induced [20] or glycerol-induced nephrotoxicity [21].

Quinacrine was also previously reported to have an antioxidant effect in ulcerative colitis [22], status epilepticus [23] and neurodegenerative diseases [24]. In context, our results showed that quinacrine plays an antioxidant role by blocking cisplatin-induced oxidative stress in kidney. This was demonstrated by a significant decrease in renal MDA concentration and an increase in renal TAC. Furthermore, we studied the role of quinacrine in modulating SIRT-1 and its underlying inflammatory and apoptosis pathways. Our data has shown that cisplatin caused a significant downregulation of renal tissue levels of SIRT-1, which was reversed by quinacrine. These results confirm the regulatory role of SIRT-1 in drug-induced renal injuries [25]. While the modulatory effect of cisplatin of SIRT-1 was previously reported [9,10,11,26,27], this work is the first, to the best of our knowledge, to report the effect of quinacrine on SIRT-1 regulation.

As previously shown, inflammation plays a major role in the pathogenesis of cisplatin-induced renal injury [28,29]. Consistent with previous work [29,30,31], our results showed that cisplatin evoked a marked pro-inflammatory response, which was indicated by the significant increase in renal levels of inflammatory mediators ICAM-1 and TNF-α. Importantly, quinacrine successfully reduced cisplatin-upregulated levels of ICAM-1 and TNF-α. These results were consistent with different studies that reported the inhibitory effect of quinacrine on ICAM-1 [32] and TNF-α expression [33].

Recent studies revealed that SIRT-1 upregulation alleviates apoptosis in drug-induced injuries [34,35,36]. Apoptosis causes DNA fragmentation, which leads to increased oxidative stress and subsequent inflammation [34,37]. p53, a sensor of DNA damage and cell death, promotes apoptosis by regulating expression of caspases enzymes and BCL2 family members, all of which were reported to highly modulate cisplatin-induced apoptosis [28,38]. Among caspases, caspase-8 is an important mediator for an inflammatory cell death process known as necroptosis [39,40].

In this study, cisplatin led to a significant increase in expression levels of caspases, BAX and p53 with a significant decrease in expression levels of BCL2. These results suggest that SIRT-1 downregulation might play a regulatory role in cisplatin-induced nephrotoxicity. Consistently, Kim et al. (2019) stated that SIRT-1 activation attenuates cisplatin-induced cell apoptosis, probably through deacetylating p53 [11]. Quinacrine blocked cisplatin-induced apoptosis by upregulating SIRT-1. Quinacrine has also been reported to have anti-apoptotic effects both in vitro [41] and in vivo [42]. Indeed, our data suggest that quinacrine inhibits cisplatin-induced apoptosis and necroptosis processes. Further investigation is needed to establish whether the inhibitory effect of quinacrine on both apoptosis and necroptosis is synergistic. The impact of such synergy was shown to be effective in alleviating cisplatin-induced nephrotoxicity [43].

Altogether, cisplatin downregulated SIRT-1 levels, which might be—in part—due to induction of oxidative stress. SIRT-1 downregulation resulted in an increase in pro-inflammatory markers (ICAM-1 and TNF-α) in addition to upregulation of p53 and pro-apoptosis proteins (BAX and caspases). It also led to downregulation of the anti-apoptosis protein BCL2. Quinacrine reversed these effects, which efficiently protected against cisplatin-induced nephrotoxicity (Figure 4C).

## 4. Materials and Methods

### 4.1. Animals

Twenty-nine male Sprague Dawley rats weighing (180–200 gm) were purchased from the Faculty of Pharmacy, Mansoura University, Egypt. Rats were allowed to be acclimated by keeping them under standard conditions of a 12 h dark and 12 hr light cycle, 23–27 °C temperature, and 48–52% humidity for a week before administration of the first dose. Rats were provided with the standard pellet diet and water ad libitum. The animal care and experiments described in this study complied with “Research Ethics Committee” Faculty of Medicine, Mansoura University, Mansoura, Egypt, which is in accordance with “Principles of Laboratory Animal Care” (NIH publication No. 85-23, revised 1985) in animal care and experiments.

### 4.2. Study Medications

Cisplatin (232120) and quinacrine (Q3251) were purchased from Sigma-Aldrich (Saint Louis, MO, USA). All other chemicals used in the study were of high purity and analytical grade.

### 4.3. Induction of Cisplatin Toxicity

Cisplatin was dissolved in saline. Acute renal toxicity was induced by intraperitoneal injection of 10 mg/kg cisplatin, a single dose, at day 5 [16,44].

### 4.4. Experimental Model

Rats were randomly divided into three groups:

1. Normal group (8 rats): rats were injected with 0.2 mL saline intraperitoneally, daily for 10 days.
2. Cisplatin group (13 rats): rats were intraperitoneally injected with 0.2 mL saline daily for 10 days, except at day 5, when they were injected with cisplatin single-dose intraperitoneally (10 mg/kg).
3. Cisplatin + quinacrine (8 rats): rats were intraperitoneally injected with quinacrine (10 mg/kg/day) for 10 days [20,21,42,45] and were injected with a cisplatin single dose (10 mg/kg) at day 5 an hour after quinacrine treatment.

The mortality of the rats was daily recorded (Supplementary Table S1). At the end of the model, twenty-four hours after the last quinacrine injection, the rats were weighed. Blood samples (5 mL) were withdrawn via retro-orbital puncture under light ether anesthesia. Blood samples were allowed to coagulate, centrifuged, and then serum was aliquoted for further biochemical analyses.

Rats were sacrificed to dissect the kidneys. The kidneys were rinsed with cold phosphate buffer saline (PBS) pH:7.4, blotted dry with filter paper, and then weighed. A transverse cut of the right kidney was fixed in 10% phosphate-buffered formalin (PBF) for histopathological and immunohistochemical analyses. The left kidney was homogenized in cold PBS, centrifuged, and then the supernatant was aliquoted and kept at −80°C for further oxidative stress and ELISA analyses.

### 4.5. Serum Biochemical Analysis

Serum was used for the determination of creatinine (#10053) and urea (#10505) concentrations, according to Bartles et al., [46] and Fawcett el al., [47], respectively, using commercially available colorimetric kits (Human Co, Wiesbaden, Germany).

### 4.6. Renal Oxidative Stress Analysis

A supernatant of the kidney tissue homogenates was used for determination of the MDA (# MD 25 29) concentration, following the method of Kei et al. [48] and TAC (# TA 25 13), following the method of Koracevic et al. [49], using commercially available colorimetric kits (Biodiagnostic company, Cairo, Egypt).

### 4.7. Renal ELISA Analysis

A supernatant of the kidney tissue homogenates was used for determination of SIRT-1, ICAM-1, and TNF-α concentrations, according to the manufacturer’s instructions, using the following ELISA kits: CSB-EL021339RA, CSB-E04576r and CSB-E11987r, respectively (Cusabio, Wuhan, China). Briefly, standards and samples were added to a 96-well plate, covered with adhesive strip, and incubated for 2 h at 37 degrees. Liquids were removed from all wells without washing. Biotin-antibody (1×) was added to each well, covered with a new adhesive strip and incubated for 1 h at 37 °C. The antibody was removed and the wells were washed three times, using the provided washing buffer. HRP-avidin (1×) was added to each well, and the microtiter plate was covered with a new adhesive strip and incubated for 1 h at 37 °C. The solution was aspirated, and the wells were washed five times. TMB substrate was added to each well and incubated for 20 min at 37 °C in dark. STOP Solution was added to each well, and the optical density was measured, using a microplate reader at 450 nm.

### 4.8. Histopathological and Immunohistochemical Analyses

The formalin-fixed kidney tissues were embedded in paraffin. Sections were cut into 5 μm in thickness and stained with hematoxylin and eosin (H&E). Slides were used for renal morphological and structural alteration examination under light microscopy. Pictures were captured, using a digital camera. The slides were blindly examined.

For % area of fibrosis, the sections were stained with Masson’s trichrome for examining the fibrosis formation and assessment of % area of fibrosis, as Masson’s trichrome stains fibrotic areas with blue and parenchymal cells with red.

For immunohistochemical analysis, the sections were deparaffinized, rehydrated, and immersed in antigen retrieval solution (EDTA solution, pH 8). The sections were treated with 3 % hydrogen peroxide and protein block, followed by overnight incubation at 4 °C with the following antibodies: TNF-α (A0277: ABclonal, Woburn, MA, USA), Caspase-1 (sc-392736: Santa Cruz Biotechnology, INC., Heidelberg, Germany), Caspase-3 (GB11532: servicebio, Wuhan, China), Caspase-8 (CBS-PA001234: Cusabio, Wuhan, China), Caspase- 9 (CSB-PA001235: Cusabio, Wuhan, China), BAX (GB11007-1: Servicebio, Wuhan, China), BCL2 (61-0005-2: Genemed, San Francisco, CA, USA) and p53 (A11232: ABclonal, Woburn, MA, USA) at a 1:100 dilution factor. The slides were rinsed three times with PBS, and incubated with appropriate secondary antibodies for 30 min at 25 °C. As a negative control, the primary antibody was replaced by normal rat serum. The specificity of the used antibodies was checked using No antibody control, secondary anti-mouse and secondary anti-rabbit controls are showing negative staining to confirm that the reported signal was antigen specific (Supplementary Figure S2). For the analysis of antibody binding, a diaminobenzidine kit was used against the H&E counterstain. Finally, the slides were checked using light microscopy to detect the distinct brown-colored reaction.

The slides for Masson’s trichrome and immune-histochemical stainings were photographed, using an Olympus^®^ digital camera installed on an Olympus^®^ microscope with 1/2 X photo adaptor, using 400× objective. The images were then analyzed on an Intel^®^ Core I3^®^ based computer, using VideoTest Morphology^®^ software (St.-Petersburg, Russia) with a specific built-in routine for area, % area measurement and object counting. Masson’s trichrome blue color stained areas, Caspase-1, -3, -8, and -9, and BCL2 and p53 brown color immunostained areas were blindly determined, using ImageJ software (National Institutes of Health, Bethesda, MD, USA), taking six reads for each section at 400× magnification. Briefly, digital image analysis was performed by uniformly adjusting size of all images to 12.7 centimeters in width and 9 centimeters in length (300 dpi). In this case, a scale bar was set to (25 microns). All images were loaded into the (image J) program, where the 6 fields were investigated. Finally, the average area and % area for each group were calculated relative to the control normal group.

### 4.9. Statistical Analysis

All data are presented as mean ± standard error of the mean (S.E.M). Statistical analysis was performed via GraphPad Prism V 5.02 (GraphPad Software Inc., San Diego, CA, USA). The distribution of data was analyzed to check the parametric or non-parametric tests, which will follow. One-way ANOVA followed by Bartlett’s test (post-test) was used to determine the statistical significance between groups. *p* ≤ 0.05 was considered statistically significant, with the following used symbols: * *p* < 0.05; ** *p* < 0.01; *** *p* < 0.001.

## 5. Conclusions

The current study proposes, for the first time, that quinacrine ameliorated cisplatin-induced renal toxicity via upregulation of SIRT-1. Moreover, no previous studies, to our best knowledge, have reported the effect of quinacrine on SIRT-1 regulation. Furthermore, quinacrine significantly downregulated the pro-inflammatory proteins (ICAM-1 and TNF-α), restored the cisplatin-disrupted oxidative balance, suppressed the apoptotic mediators (caspases, BAX, and p53), and upregulated the anti-apoptotic protein BCL2. Although clinical studies are needed to establish the renoprotective effect of quinacrine against cisplatin-induced renal toxicity, this study provides evidence for a potential novel therapeutic use of quinacrine as a protective agent against cisplatin-induced renal injury.

## Data Availability

The data that support the findings of this study are available from the authors upon reasonable request.

## Supplementary Materials

The following are available online at https://www.mdpi.com/article/10.3390/ijms221910660/s1, Figure S1: Quinacrine attenuated cisplatin-induced apoptosis., Figure S2: The specificity of the used antibodies for immunohistochemistry. Table S1: The number of rats in each group at day 0 (the start) and day 10 (the end) of the experimental model.
