# Supplementary material for: Quinacrine Ameliorates Cisplatin-Induced Renal Toxicity via Modulation of Sirtuin-1 Pathway
Source: Int J Mol Sci. 2021 Oct 1;22(19):10660. doi: 10.3390/ijms221910660 (PMC8508772; doi:10.3390/ijms221910660)
Supplement: Supplementary file 1 [file ijms-22-10660-s001.zip › ijms-1377699-supplementary.pdf]

Supplementary Data:

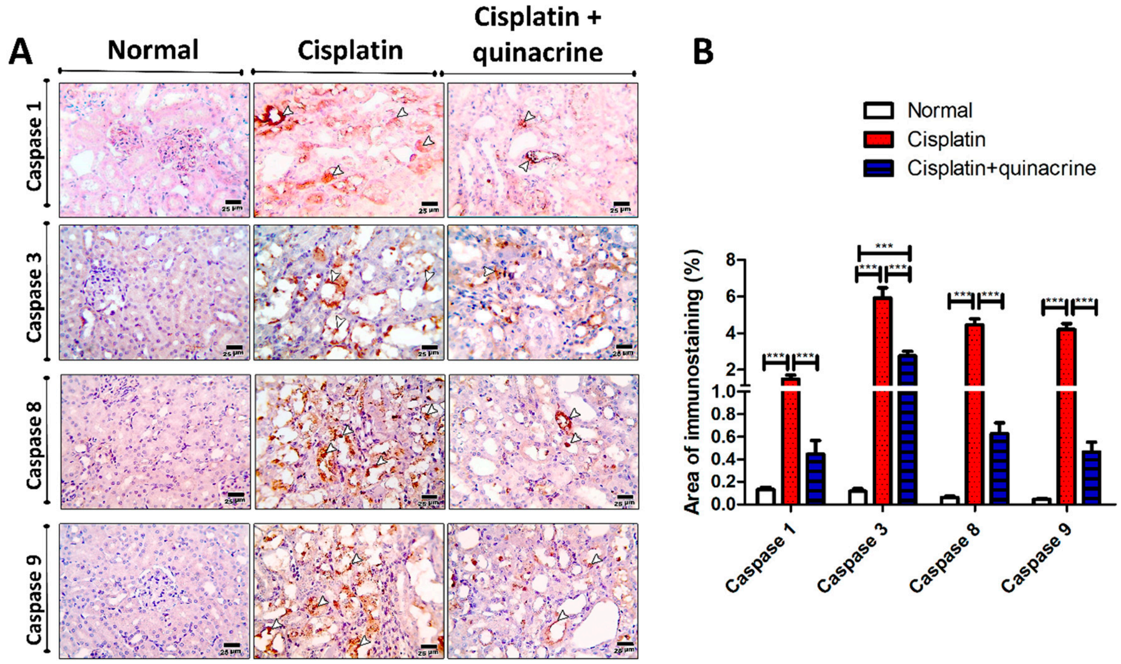

Supplementary Figure S1: Quinacrine attenuated cisplatin-induced apoptosis. A) Microscopic pictures of immunostained renal sections against caspases 1,3, 8 & 9; Arrowheads point to positive brown reaction; High magnification X:400 bar 25. B) area of immunostaining of caspases 1,3,8 & 9 (%) (n=6). ***P<0.001. Data are expressed: mean±SEM

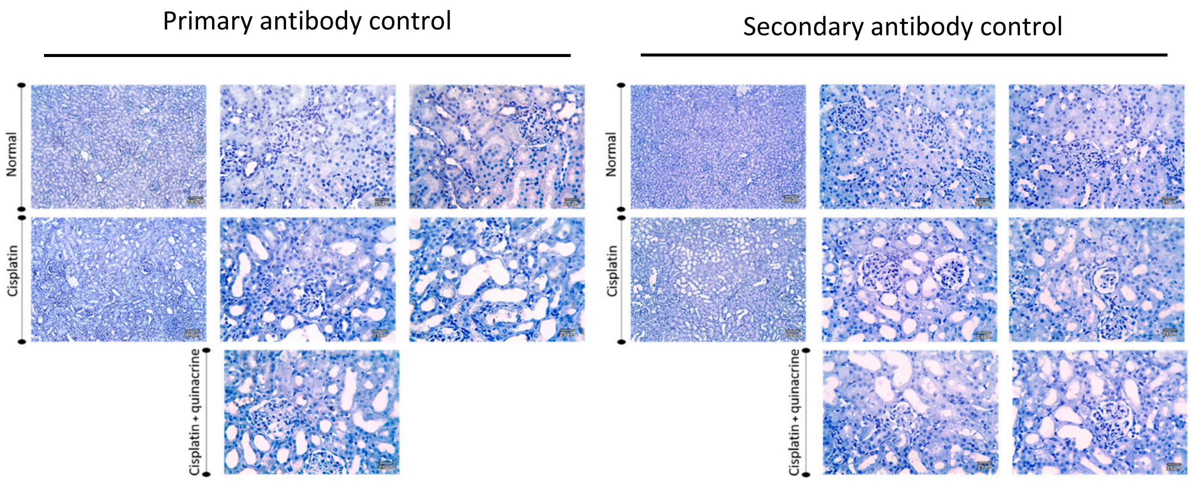

Supplementary Figure S2: The specificity of the used antibodies for immunohistochemistry. No antibody control, secondary anti-mouse and secondary anti-rabbit controls are showing negative staining which confirms that the reported signal was antigen specific. High magnification X:400 bar 25

Supplementary table S1:

The number of rats in each group at day 0 (the start) and day 10 (the end) of the experimental model

Group name/Day	D 0	D1	D2	D3	D4	D5	D6	D7	D8	D9	D10
Normal group	n=8	n=8	n=8	n=8	n=8	n=8	n=8	n=8	n=8	n=8	n=8
Cisplatin group	n=13	n=13	n=13	n=13	n=13	n=13	n=12	n=12	n=12	n=10	n=7
Cisplatin + quinacrine group	n=8	n=8	n=8	n=8	n=8	n=8	n=8	n=8	n=8	n=8	n=7
